# Comparative analysis of replication and immune evasion among SARS-CoV-2 subvariants BA.2.86, JN.1, KP.2, and KP.3

**DOI:** 10.1128/mbio.03503-24

**Published:** 2025-04-29

**Authors:** Yanping Hu, Jing Zou, Michael D. Nguyen, Hope C. Chang, Jason Yeung, Haiping Hao, Pei-Yong Shi, Ping Ren, Xuping Xie

**Affiliations:** 1 Department of Microbiology and Immunology, University of Texas Medical Branch 547647, Galveston, Texas, USA; 2 Department of Pathology, University of Texas Medical Branch 198642, Galveston, Texas, USA; 3 Department of Biochemistry and Molecular Biology, University of Texas Medical Branch 198643, Galveston, Texas, USA; 4 Sealy Institute for Drug Discovery, University of Texas Medical Branch 12338, Galveston, Texas, USA; Medical School, National and Kapodistrian University of Athens, Athens, Greece

**Keywords:** SARS-CoV-2, immune evasion, BA.2.86, JN.1, KP.2, KP.3, viral fitness

## Abstract

The severe acute respiratory syndrome coronavirus 2 (SARS-CoV-2) BA.2.86 sublineage and its descendants (JN.1, KP.2, and KP.3) have acquired key recurrent mutations (R346T, L455S, F456L, and Q493E) and became predominant strains, following the epidemiological progression: BA.2.86→JN.1→KP.2→KP.3. However, the mechanisms driving this succession remain incompletely understood. In this study, we assessed the replication fitness of SARS-CoV-2 strains containing spike sequences from BA.2.86 and its descendants (JN.1, KP.2, and KP.3) in primary human airway epithelium cells and their sensitivity to neutralization by human sera. Our analysis revealed reduced spike cleavage in JN.1 and KP.2 virions compared to BA.2.86 and KP.3, indicating that receptor-binding domain (RBD) mutations L455S and Q493E, despite being distant from the furin cleavage site, can influence spike cleavage. JN.1, with the additional L455S mutation, replicated more slowly than BA.2.86 but was more resistant to neutralization by XBB.1.5-infection sera, suggesting that immune evasion driven by the L455S mutation is the primary factor behind the BA.2.86-to-JN.1 transition. KP.2, carrying additional R346T, L455S, and F456L mutations, showed both enhanced replication and increased resistance to neutralization by JN.1-infection sera, indicating that the combined effects of these mutations on immune evasion and viral fitness drive the JN.1-to-KP.2 shift. The latest strain, KP.3, derived from JN.1 with the L455S, F456L, and Q493E mutations, demonstrated even greater replication than KP.2 while maintaining similar neutralization sensitivity to JN.1-infection sera, suggesting that Q493E further enhances viral replication and drives the KP.2-to-KP.3 transition. These findings highlight how specific recurrent spike mutations in BA.2.86 descendants fine-tune viral replication fitness and immune evasion, promoting their emergence and dominance.

**IMPORTANCE:**

The study advances our understanding of the roles of immune evasion and replication fitness in driving the evolution of severe acute respiratory syndrome coronavirus 2 (SARS-CoV-2) from the BA.2.86 sublineage to its descendants (JN.1, KP.2, and KP.3). Through head-to-head comparisons of the replication fitness of recombinant SARS-CoV-2 strains containing spike sequences from BA.2.86 and its descendants in primary human airway epithelium cells, alongside assessments of their neutralization sensitivity to human sera, we revealed how recurrent mutations R346T, L455S, F456L, and Q493E in the receptor-binding domain (RBD) fine-tune immune evasion and viral replication fitness, underscoring the critical need for updated countermeasures to combat newly emerged SARS-CoV-2 variants. Additionally, our analysis showed that the L455S and Q493E mutations in the RBD can influence spike cleavage, offering new insights into SARS-CoV-2 spike biology.

## INTRODUCTION

**Fig 1:**
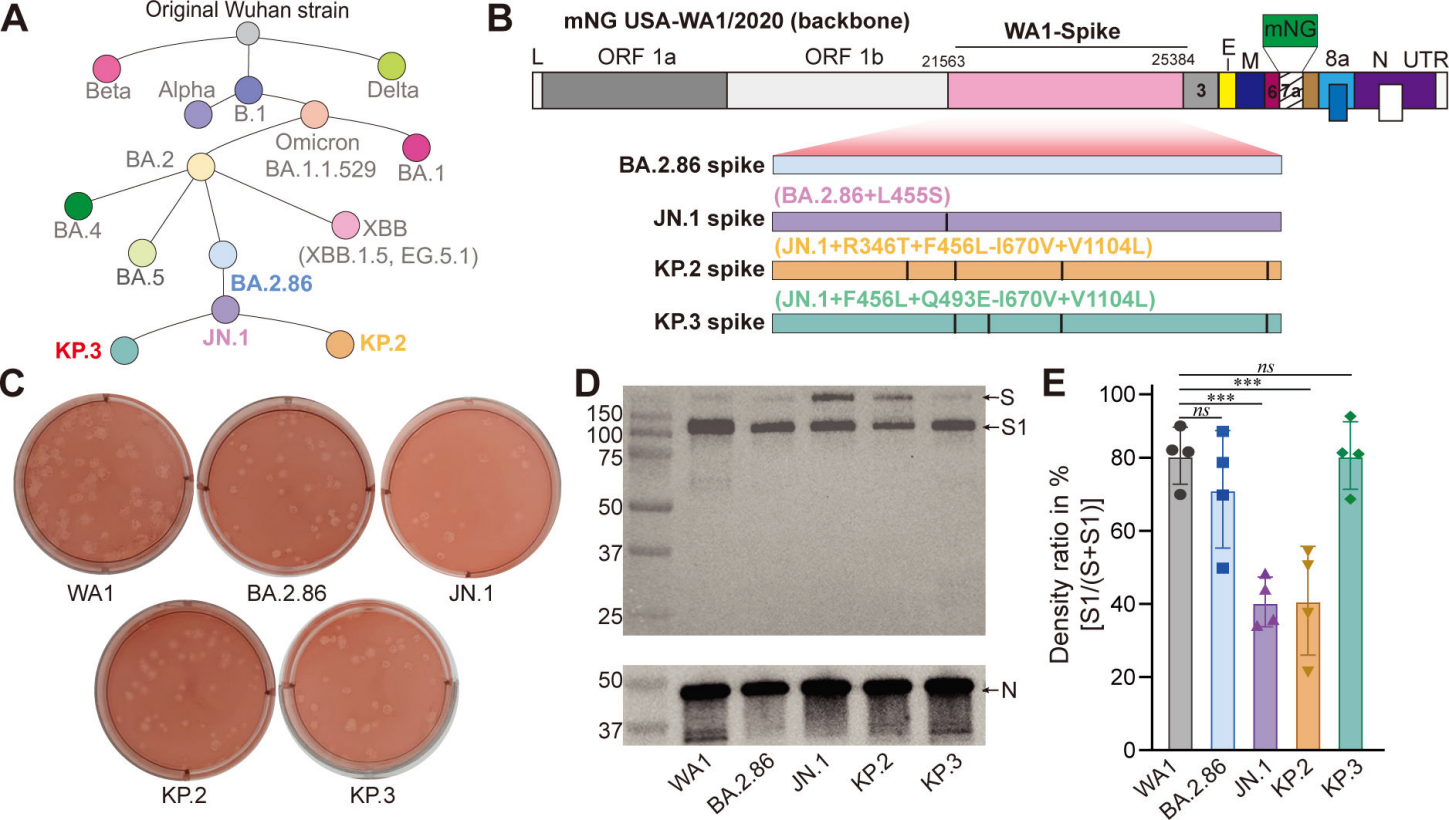
Generation and characterization of mNG SARS-CoV-2 spike variants. (**A**) Phylogenetic relationship of BA.2.86 and its descendants, based on the data from COVID Data Tracker (5). (**B**) Schematic of mNG SARS-CoV-2 spike variant construction. The mNG SARS-CoV-2 derived from strain USA-WA1/2020 (WA1) was used as a backbone. The spike gene of WA1 was substituted by that from each variant. L, leader sequence; ORF, open reading frame; E, envelope protein; M, membrane protein; N, nucleocapsid; UTR, untranslated region; mNG, mNeonGreen. (**C**) Plaque morphologies of mNG SARS-CoV-2 variants on Vero E6-TMPRSS2 cells. (**D**) Western blot analysis of spike and nucleocapsid protein in virions. S, full-length spike; S1, N-terminal furin cleavage fragment of spike. (**E**) Ratio of cleaved spike (S1) to total spike (S plus S1) in virions. The means and standard deviations from four independent experiments are shown. ***P < 0.001 (determined using one-way ANOVA followed by Dunnett’s multiple comparison test), statistical significance; ns, no statistical significance.

Severe acute respiratory syndrome coronavirus 2 (SARS-CoV-2), the viral pathogen responsible for the coronavirus disease 2019 (COVID-19) pandemic, has caused immense global morbidity and mortality since its outbreak in December 2019 (1). Enhanced immune evasion and viral transmissibility in human populations have driven the continuous evolution of SARS-CoV-2. This has led to successive waves of global infections by diverse variants, including Alpha, Beta, Delta, and Omicron. The emergence of Omicron marked a significant milestone in SARS-CoV-2’s evolutionary trajectory. Compared to the original Wuhan strain, Omicron (BA.1) contains over 30 amino acid mutations in its spike protein, contributing to its greatly enhanced transmissibility and immune evasion (2–4). Omicron has since diversified into various sublineages, such as BA.2, BA.4/5, XBB, and its descendants (XBB.1.5 and EG.5.1) (Fig. 1A), each triggering surges of widespread infections.

BA.2.86, another notable sublineage derived from BA.2, carries over 30 amino acid changes in its spike protein compared to both BA.2 and XBB.1.5, a degree of change similar to the leap between Omicron BA.1 and the original Wuhan strain (6). Since its detection in July 2023, BA.2.86 and its descendants have steadily spread worldwide, displacing the previously dominant XBB.1.5 and EG.5.1 and other subvariants containing L455F and F456L (commonly referred to as “FLip”) mutants (7, 8). The exact mechanisms underlying BA.2.86’s rise over prior variants remain unclear. Epidemiological data suggest that BA.2.86 possesses higher transmissibility in human populations than EG.5.1 (9), potentially due to increased angiotensin-converting enzyme 2 (ACE2) binding affinity (10, 11). However, BA.2.86 has not demonstrated a significant advantage in immune evasion of humoral immunity or replication in cell cultures and animal models compared to EG.5.1 (6, 12–16). Following this, JN.1 rapidly emerged from BA.2.86 through a single L455S mutation in the receptor-binding domain (RBD), enhancing immune evasion and transmissibility in human populations (13, 17). By January 2024, JN.1 had become the dominant strain in many countries. JN.1 subsequently gave rise to further descendants, including KP.2 and KP.3, which emerged concurrently in March 2024. KP.2, featuring additional R346T and F456L (known as “FLiRT”) mutations in the spike protein, replaced JN.1 by May 2024. KP.3, which has the additional Q493E mutation but lacks R346T, overtook KP.2 as the dominant strain by early June 2024 (18).

Epidemiological studies indicate that the successive rise in transmissibility from BA.2.86 to JN.1, and then KP.2 or KP.3, was largely driven by enhanced immune evasion and increasing transmissibility. However, most of the immune evasion and viral infectivity analysis has been conducted using pseudoviruses (9, 13, 14, 18, 19). The role of viral fitness and immune evasion in the epidemiological transition of BA.2.86→JN.1→KP.2→KP.3 has been inadequately characterized in the context of infectious SARS-CoV-2.

In this study, we constructed highly attenuated SARS-CoV-2s incorporating spike sequences from BA.2.86 and its descendants (JN.1, KP.2, and KP.3). We utilized a pairwise competition assay to compare their replication fitness in primary human airway epithelium (HAE) cell culture. Additionally, we assessed their sensitivity to neutralization by human sera using a live SARS-CoV-2 neutralization assay. Through this, we aim to understand the roles of recurrent mutations in shaping the evolutionary trajectory of BA.2.86 and its descendants.

## MATERIALS AND METHODS

### Cells

Vero E6 (ATCC CRL-1586) was purchased from the American Type Culture Collection (ATCC, Bethesda, MD) and maintained in high-glucose Dulbecco’s modified Eagle’s media (DMEM) containing 10% fetal bovine serum (FBS; HyClone Laboratories, South Logan, UT) and 1% penicillin/streptomycin at 37°C with 5% CO_2_. Vero E6 cells expressing TMPRSS2 (JCRB1819) were obtained from SEKISUI XenoTech, LLC, and grown in the same culture media as Vero E6 cells with the addition of 0.5 mg/mL G418. Cells, culture media, and antibiotics were purchased from Thermo Fisher Scientific (Waltham, MA). Both cell lines tested mycoplasma negative.

### Human serum

Two panels of human sera collected at UTMB were used in the study. The first sample panel consisting of 61 sera was collected 15–117 days (median 45) after XBB.1.5 infection (as determined by reverse transcription PCR [RT-PCR]) from individuals aged 15–85 years old (median 46) who had received two to four doses of mRNA vaccines. Nineteen of them had also received a BA.5 bivalent booster. However, the infection history before XBB.1.5 infection in individuals was unavailable. The second sample panel consisting of 41 sera was collected 20–111 days (median 45) after JN.1 infection (as determined by RT-PCR) from individuals aged 7–84 years old (median 46). The vaccine and infection history of the second panel was not determined since most of the population had been vaccinated or infected. Patient information was completely deidentified from all specimens. The deidentified human sera were heat-inactivated at 56°C for 30 min before the neutralization test. The serum information is presented in Tables S1 and S2.

### Generation of recombinant mNeonGreen (mNG) SARS CoV-2 BA.2.86-spike variants

Recombinant BA.2.86-, JN.1-, KP.2-, and KP.3-spike mNG SARS-CoV-2s were constructed by engineering the complete *spike* gene from the indicated strains into an infectious cDNA clone of mNG USA-WA1/2020 (20, 21). The full-length infectious cDNA clone of SARS-CoV-2 was assembled by *in vitro* ligation. Subsequently, the full-length viral RNA was synthesized through *in vitro* transcription. The RNA transcripts were electroporated in Vero E6-TMPRSS2 cells to recover the recombinant viruses. Supernatants (P0) were harvested 2–3 days after electroporation. P0 stock was further passaged once on Vero E6 cells to produce P1 stock. All virus preparation and neutralization assays were carried out at the biosafety level 3 (BSL-3) facility at the University of Texas Medical Branch at Galveston. All recombinant viruses were verified by Sanger sequencing or next-generation sequencing (NGS) to ensure the absence of undesired mutations in their genome.

### Fluorescent focus reduction neutralization test (FFRNT)

Neutralization titers of human sera were measured by FFRNT using BA.2.86-, JN.1-, KP.2, and KP.3-spike mNG SARS-CoV-2s using a previously established FFRNT protocol (22). Briefly, 2.5 × 10^4^ Vero E6 cells per well were seeded in 96-well plates (Greiner Bio-one). The cells were incubated overnight. On the next day, each serum was twofold serially diluted in the culture medium with the first dilution at 1:20 (final dilution range of 1:20 to 1:20,480). The diluted serum was incubated with 100–150 fluorescent focus units (FFUs) of mNG SARS-CoV-2 at 37°C for 1 h, after which the serum virus mixtures were loaded onto the pre-seeded Vero E6 cell monolayer in 96-well plates. After 1 h infection, the inoculum was removed, and 100 µL of overlay medium (supplemented with 0.8% methylcellulose) was added to each well. After incubating the plates at 37°C for 16 h, raw images of mNG foci were acquired using a Cytation 7 (BioTek) armed with a 2.5× FL Zeiss objective with a wide-field of view and processed using the Gen5 software settings (green fluorescent protein [GFP] [469,525], threshold 4,000 and object selection size 50 μm–1,000 μm). Foci in each well were counted using the Gen5 software and normalized to non-serum-treated controls to calculate relative infectivities. The FFRNT_50_ value was defined as the minimal serum dilution that suppressed >50% of fluorescent foci. The neutralization titer of each serum was determined in duplicate assays, and the geometric mean was taken. Tables S2 to S4 summarize the FFRNT_50_ results. Data were initially plotted in GraphPad Prism 9 software and assembled in Adobe Illustrator. FFRNT_50_ of <20 was treated as 10 for plotting and statistical analysis.

### Pairwise competition experiment

HAE cell cultures, EpiAirway, were purchased from MatTek Life Sciences. This primary 3D mucociliary tissue model consists of normal, human-derived tracheal/bronchial epithelial cells. Two mNG SARS-CoV-2 spike variants were mixed in a 1:1 ratio based on viral titers in PFU/ml, determined by plaque assay on Vero E6-TMPRSS2 cells. The inoculum was prepared in Dulbecco's phosphate-buffered saline (DPBS) containing virus mixture with each virus at a final concentration of 1 × 10^6^ PFU/mL. An aliquot of the inoculum was also stored to determine the ratio of input viruses. Before infection, HAE cultures were bathed in DPBS at 37°C for 30 min. After removing the DPBS, 200 µL of inoculum per well was added at the apical side. After 2 h incubation at 37°C, 5% CO_2_, the inoculum was removed, and cells were washed with DPBS three times to remove unbound viruses. At each time point, 300 µL of DPBS was added to the apical side of HAE and incubated at 37°C, 5% CO_2_, for 30 min to elute viruses. DPBS washes containing viruses were then collected into a 2 mL tube. On day 3 post-infection, after collecting DPBS washes, 300 µL of Trizol reagent (Invitrogen) was added to the cells. Cell lysates were harvested to isolate total cellular RNAs. All samples were stored in a −80°C freezer before use.

Upon analysis, 100 µL of each sample was mixed with a fivefold volume of Trizol LS reagent (Thermo Fisher Scientific). Viral RNAs were extracted using the Direct-zol RNA Miniprep Plus RNA kit (Zymo Research) according to the manufacturer’s instructions. cDNA amplicons with 100–200 base pairs containing unique mutations from each strain were generated using SuperScript IV One-Step RT-PCR System with primer pairs. Primer set 1, 455–456-F (sequence: 5′-AGGCTGCGTTATAGCTTGGAATT-3′) and 455–456-R (sequence: 5′-GGCCTGATAGATTTCAGTTGA-3′), was used to amplify cDNA encoding amino acids from position 429 to 472 of the *spike* gene containing unique differences between BA.2.86, JN.1, and KP.2. Primer set 2, 346-F (sequence: 5′-AACAGAATCTATTGTTAGATTTCCTAATGTTACAAACT TGTGCCCTTTT-3′) and 346-R (sequence: 5’TAGGACAGAATAATCAGCAACACAGTTGCTGAT TCTCGTCCTGTTCCAAGCATAAACAG-3′), was used to amplify the cDNA encoding amino acids from position 319 to 365 of the *spike* gene containing unique mutations differentiating KP.2 from KP.3. The cDNA fragments were gel-purified and sent for Illumina NGS at the sequencing core facility at UTMB.

### Virion purification and Western blot

One milliliter of each virus from the P1 stocks harvested from Vero E6 cells was mixed with polyethylene glycol-8000 (Sigma-Aldrich) at a final concentration of 10%. The mixtures were incubated at room temperature for 30 min followed by centrifugation at 4,000 × *g* for 10 min to pellet down the virions. The pellets were washed once with 70% ethanol and resuspended in 2× Laemmli sample buffer containing 0.7 M of β-mercaptoethanol. The samples were heat-inactivated at 95°C for 15 min and analyzed on a 4–20% gradient SDS-PAGE gel. Viral spike and nucleocapsid proteins were detected by Western blot using specific antibodies anti-S1 (Sino Biological #40591-T62) and anti-N (Sino Biological #40143-R001), respectively. The densitometry analysis was performed using ImageJ.

### Plaque assay

Approximately 1.0 × 10^6^ Vero E6-TMPRSS2 cells per well were seeded into six-well plates and cultured at 37°C, 5% CO_2_, for 16 h. Virus samples were 10-fold serially diluted in DMEM with 2% FBS. A total of 200 µL of each dilution was added to cell monolayers. After 1 h of incubation at 37°C, 5% CO_2_, the inoculum was removed. Two milliliters of overlay medium containing DMEM with 2% FBS, 1% penicillin/streptomycin, and 1% Seaplaque agarose (Lonza, Walkersville, MD) were added to each well. After incubation at 37°C with 5% CO_2_ for 2 days, 2 mL of overlay medium supplemented with neutral red (Sigma-Aldrich, St. Louis, MO) was added to each well to stain the cells. The next day, plaques were counted and titers were calculated.

### Statistics and reproducibility

No statistical method was used to predetermine the sample size. The serum samples were collected based on availability. No data were excluded from the analyses. The experiments were not randomized. Patient information was blinded in the study. The investigators were blinded to sample identity during data collection and/or analysis. The neutralization experiments were performed in duplication. All attempts at replication were successful. Continuous variables were summarized as the geometric mean with 95% confidence intervals or median. Sera with undetectable (<20) antibody titers were assigned an antibody titer of 10, for purposes of geometric mean titer (GMT) calculations or statistical comparisons. Comparison between neutralization titers was performed using a Wilcoxon matched-pairs signed-rank test using GraphPad Prism 10.

For competition experiments, five independent infections were performed for each group. Simple linear regression was performed to analyze the statistical significance of the RNA ratio at each indicated time point versus the input RNA ratio. Absolute *P*-values were provided. *P* < 0.05 was considered statistically significant. Images were assembled using Adobe Illustrator.

## RESULTS

### Generation and characterization of SARS-CoV-2 mNG spike variants

BA.2.86 and its descendants JN.1, KP.2, and KP.3 display antigenicity that is phylogenetically distinct from XBB lineages (Fig. 1A). In this study, we focus on understanding how additional spike mutations among BA.2.86’s descendants (JN.1, KP.2, and KP.3) impact viral replication and immune evasion. To achieve this, we engineered the complete *spike* gene from BA.2.86, JN.1, KP.2, and KP.3 into the mNG SARS-CoV-2 backbone (Fig. 1 and Fig. S1). The mNG SARS-CoV-2, derived from the USA-WA1/2020 (a strain isolated in Washington State in January 2020), is substantially attenuated *in vivo* due to the insertion of an mNG reporter at the open reading frame 7 of the viral genome (21, 23). This attenuated virus has been safely used within BSL-3 facilities for neutralization studies (15, 24). Importantly, mNG SARS-CoV-2 retains robust infectivity in human primary airway epithelium cell cultures (25), a relevant *in vitro* cell culture model for studying Omicron variant infections (26). During the early outbreak, two BA.2.86 variants were identified, one with an I670V mutation and another without it. I670V does not contribute to the neutralization evasion of BA.2.86 (11). The I670V mutation was initially constructed in the BA.2.86 (15) and JN.1 constructs for this study. However, it was excluded from the KP.2 and KP.3 constructs due to its infrequency in later strains.

During the viral rescue, all mNG SARS-CoV-2 spike variants showed a high infectivity and robust replication, with infectious titers of all P1 virus stocks exceeding 10^7^ PFU/mL. These variants produced plaques similar in size but noticeably smaller than those of the original WA1-spike mNG SARS-CoV-2 (Fig. 1C), suggesting attenuation of the BA.2.86-derived spike variants in Vero E6-TMPRSS2 cells. To further characterize the virions, we precipitated those released outside of infected cells and examined spike (S) and nucleocapsid (N) proteins using Western blot (Fig. 1D). Notably, S1 and S showed greater variability than N across the samples, likely due to less sensitivity of the BA.2.86 variant spike to the anti-S1 antibody, which was generated using the parental Wuhan spike as the immunization antigen. Interestingly, we observed varied spike cleavage efficiencies among the tested variants (Fig. 1D and E). Specifically, approximately 70%–80% of spikes in BA.2.86 and KP.3 virions were cleaved, similar to the parental WA1 strain, whereas only about 40% of spikes in JN.1 and KP.2 virions underwent cleavage. This suggests that mutations in BA.2.86 and its descendants, likely L455S, can influence spike processing in virions in a context-dependent manner.

### JN.1 exhibits reduced replication in HAE but greater immune evasion compared to BA.2.86

**Fig 2:**
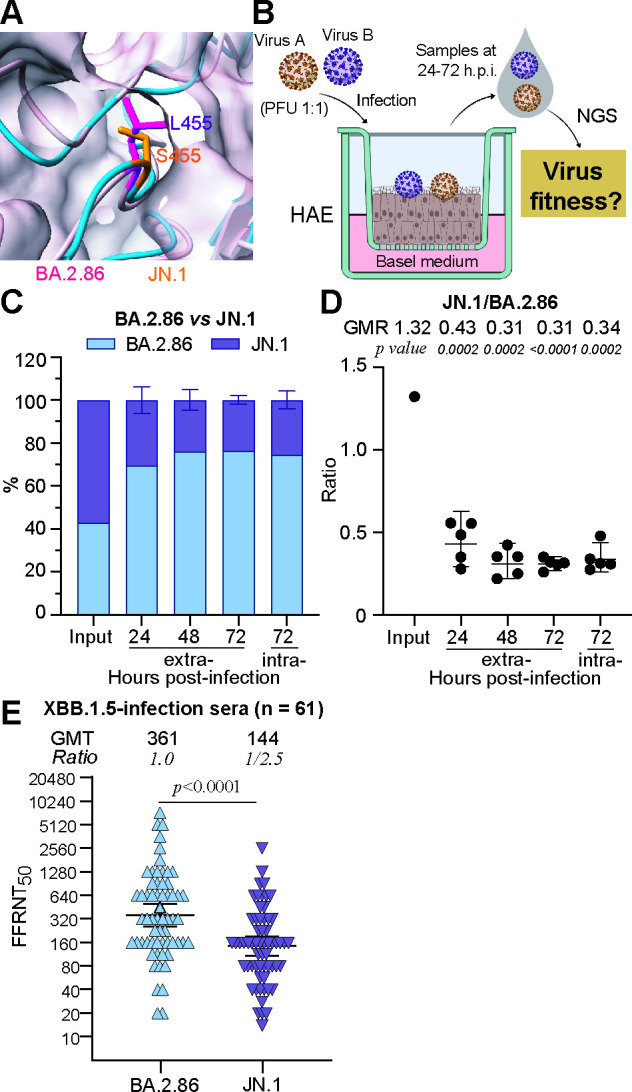
Replication and immune evasion of BA.2.86- and JN.1-spike variants. (**A**) A snapshot of the structural alignment of BA.2.86 and JN.1 RBDs. Residues leucine in BA.2.86 RBD (PDB ID:8WXL) and serine in JN.1 RBD (PDB ID:8Y5J) are highlighted. (**B**) Diagram of pairwise competition experiment. The diagram was created within BioRender.com. (**C**) The percentage of BA.2.86- and JN.1-spike RNA in infected HAE. The mean ± standard deviations from five independent experiments are shown. (**D**) Scatter plot of the ratio of BA.2.86- to JN.1-spike RNA in infected HAE. The geometric ratios (GMRs) and 95% confidence intervals (indicated as error bars) are presented. *P*-values are calculated from linear regression analysis of the RNA ratio at each given time point versus the input RNA ratio. (**E**) FFRNT_50_ of 61 human XBB.1.5-infection sera against BA.2.86- and JN.1-spike mNG SARS-CoV-2. Solid lines and numeric values above each panel indicate the GMTs. Error bars show 95% confidence intervals. The fold reduction in GMT against each variant, compared with the GMT against BA.2.86 spike, is shown. *P*-values (determined using the Wilcoxon matched-pairs signed-rank test) for group comparison are indicated.

JN.1, compared to BA.2.86, carries an additional L455S mutation within the receptor-binding motif of the spike (Fig. 2A). To address the impact of L455S on viral replication fitness, we conducted paired competition experiments with improved sensitivity to examine viral replication in primary HAE cells. Equal PFU amounts of BA.2.86 and JN.1 were mixed and used to infect the HAE. On days 1–3 post-infection, extracellular viral RNAs were harvested from DPBS washes on the apical side, and intracellular viral RNAs were collected at the end of the experiment. Viral populations were determined by NGS (Fig. 2B). Our results revealed that BA.2.86 efficiently outcompeted JN.1 in HAE (Fig. 2C and D), suggesting that the L455S mutation reduces JN.1’s fitness in human airway cultures.

We next assessed the neutralization sensitivity of JN.1 and BA.2.86 using the FFRNT assay. Sixty-one human sera (Table S1) were collected from individuals 15–117 days (median 45) after XBB.1.5-breakthrough infections (referred to as XBB.1.5-infection panel) (15). The XBB.1.5-infection panel sera neutralized BA.2.86- and JN.1-spike mNG SARS-CoV-2 with GMTs of 361 and 144, respectively (Fig. 2E). Compared to BA.2.86, the GMT against JN.1 was reduced by 2.5-fold, indicating that JN.1 is more resistant to the pre-existing humoral immunity induced by mRNA vaccines or earlier infections before its emergence. Our findings demonstrate that the additional L455S mutation in JN.1 leads to greater immune evasion compared to its predecessor strain BA.2.86, albeit with reduced viral replication fitness.

### KP.2 displays better replication in HAE and greater immune evasion compared to JN.1

**Fig 3:**
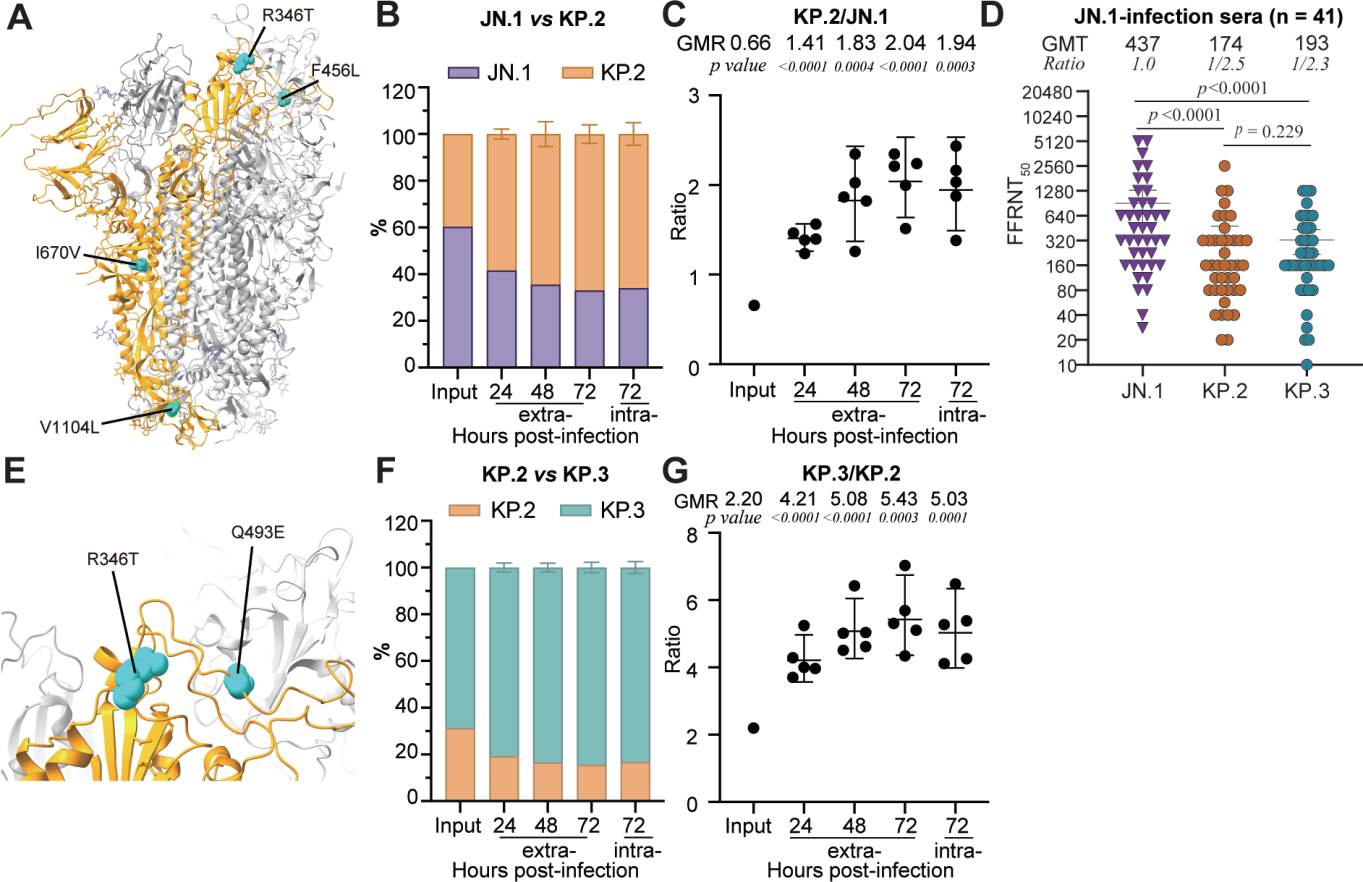
Replication and immune evasion of JN.1- versus KP.2- and KP.3- versus KP.2-spike variants. (**A**) Amino acid differences between JN.1 and KP.2 spike. Residues R346T, F456L, I670V, and V1104L are annotated in the structure of JN.1 spike (27) (PDB ID: 8Y5J). (**B**) The percentage of JN.1- and KP.2-spike RNA from infected HAE. (**C**) Scatter plot of the ratio of KP.2- to JN.1-spike RNA in infected HAE. (**D**) FFRNT_50_ of 41 human sera against JN.1-, KP.2-, and KP.3-spike mNG SARS-CoV-2. Sera were collected from individuals after JN.1 infection. Solid lines and numeric values above each panel indicate the GMTs. Error bars show 95% confidence intervals. The *P*-values (determined using the Wilcoxon matched-pairs signed-rank test) for group comparison are indicated. (**E**) Amino acid differences between KP.2 and KP.3 spikes. Residues R346T and Q493E are highlighted in the RBD structure of JN.1 (27) (PDB ID: 8Y5J). (**F**) The percentage of KP.2- and KP.2-spike RNA in infected HAE. (**G**) Scatter plot of the ratio of KP.3- to KP.2-spike RNA in infected HAE. The mean ± standard deviations from five independent experiments are shown in B and F. The geometric ratio (GMR) and 95% confidence intervals (indicated as error bars) are presented in C and G. *P*-values are calculated from linear regression analysis of the RNA ratio at each given time point versus the input RNA ratio.

KP.2 features two key mutations, R346T and F456L, in the RBD compared to JN.1 (Fig. 3A). It also differs from JN.1 by the absence of the I670V mutation in S1 and the presence of V1104L in the S2 domain. We assessed the replication fitness of JN.1 and KP.2 using similar competition experiments in HAE (Fig. 2B). Our results showed that KP.2 replicated more efficiently and outcompeted JN.1 in HAE, as evidenced by the increased KP.2-to-JN.1 RNA ratio from days 0 to 3 post-infection (Fig. 3B and C).

We also examined the sensitivities of KP.2- and JN.1-spike variants to neutralization by a panel of 41 human sera (Table S2) collected from individuals 20 to 111 days (median 45) after JN.1-breakthrough infections (referred to as JN. 1-infection panel). Pre-JN.1 vaccination or infection history was not determined due to widespread vaccination and earlier infections. The JN.1-infection panel sera neutralized JN.1- and KP.2-spike mNG SARS-CoV-2 with GMTs of 437 and 174, respectively (Fig. 3D). Compared to JN.1, the GMT against KP.2 was reduced by 2.5-fold, indicating that KP.2 has a greater evasion of humoral immunity prior to its emergence. Notably, the GMT of JN.1-infection panel sera against JN.1 was 3.0-fold higher than that of the XBB.1.5-infection panel sera (comparing Fig. 2E with Fig. 3D), suggesting that humoral immunity against JN.1 can be boosted by natural JN.1 infection or JN.1 spike-adapted vaccine.

### KP.3, compared to KP.2, exhibits enhanced replication in HAE while maintaining similar resistance to neutralization by JN.1-infection sera

The KP.3 spike differs from the KP.2 spike by lacking the R346T mutation while harboring the Q493E mutation in the RBD (Fig. 3E). Although equal PFUs were used for KP.3 and KP.2 in the input mixture for the competition experiment, NGS analysis revealed a KP.3-to-KP.2 RNA ratio of 2.20 (Fig. 3F), indicating lower infectivity of KP.3 spike than KP.2 spike in Vero E6-TMPRSS2 cells. However, in the competition experiment, the KP.3-to-KP.2 RNA ratio significantly increased to 5.43 by day 3 post-infection (Fig. 3F and G), demonstrating superior replication fitness of KP.3 in HAE.

We also evaluated the sensitivity of KP.3 spike mRNA SARS-CoV-2 to neutralization by JN.1-infection sera. As shown in Fig. 3D, JN.1-infection sera neutralized mNG SARS-CoV-2 KP.3 spike with a GMT of 193, which is 2.3-fold lower than that against mNG SARS-CoV-2 JN.1 spike. However, no significant difference was observed in the GMTs against KP.3 and KP.2, indicating that both KP.2- and KP.3-spike variants exhibit similar resistance to neutralization by JN.1-infection sera. In summary, our findings demonstrated that KP.3, compared to KP.2, exhibits enhanced viral replication fitness in primary human airway epithelial cell culture while maintaining comparable resistance to neutralization by JN.1-infection sera.

## DISCUSSION

Overall, this study reveals distinct mechanisms mediated by recurrent spike mutations in BA.2.86 descendants that drive the epidemiological transition from BA.2.86 to JN.1, then to KP.2, and finally to KP.3. The L455S spike mutation confers enhanced immune evasion, causing the rapid evolution from BA.2.86 to JN.1. The subsequent transition from JN.1 to KP.2 is driven by both improved replication fitness and immune evasion, likely due to additional R346T and F456L mutations. Furthermore, the F456L and Q493E mutations in KP.3 boost replication fitness over KP.2, facilitating the shift from KP.2 to KP.3.

Immune pressure from prior infections and vaccinations continues to shape the evolution of SARS-CoV-2. BA.2.86 descendants have accumulated two hallmark mutations, L455S and F456L, conferring strong resistance to monoclonal antibodies and humoral immunity (7, 14, 28–30). Consistent with this, our live SARS-CoV-2 neutralization analysis characterizes antigenic drift among BA.2.86-derived sublineages. JN.1 is more resistant to neutralization by sera from XBB.1.5 infections than BA.2.86. Both KP.2 and KP.3 exhibit increased resistance to neutralization by JN.1-infection sera relative to JN.1 itself. The immune evasion characteristics of JN.1, KP.2, and KP.3 have also been observed in pseudovirus systems across various immune backgrounds (9, 13, 14, 18, 19, 30–32). Notably, additional spike mutations, including R346T in KP.2, Q493E in KP.3, and V1104L in both KP.2 and KP.3, have emerged convergently but do not significantly affect serum neutralization (28). Our pairwise competition assays suggest these mutations may contribute to fine-tuning viral replication fitness. Overall, our results show a positive correlation between the fitness of spike variants in human airway epithelium cells and spike-ACE2 binding affinity. JN.1 replicates more slowly than BA.2.86, likely due to reduced ACE2 binding affinity caused by the L455S mutation (13, 32, 33). In contrast, KP.2 efficiently outcompetes JN.1 spike, consistent with its improved ACE2 binding affinity conferred by the R346T mutation (28) and the synergistic effects of L455S and F456L (34, 35). KP.3 further enhances replication fitness through the combined effects of Q493E and F456L, which significantly increase ACE2 binding affinity (34, 35). While L455S or Q493E alone significantly impairs ACE2 binding to JN.1 or XBB.1.5 RBD, and F456L has minimal effect on ACE2 binding to JN.1 RBD (28), the combinations R346T/L455S/F456L or L455S/F456L/Q493E greatly increase ACE2 binding to BA.2.86 RBD (34, 35). This suggests that context-dependent or epistatic interactions in BA.2.86 are involved in fine-tuning spike-receptor binding. These interactions may allow the acquisition of compensatory mutations to counteract the negative impact of L455S on ACE2 binding affinity while preserving the immune evasion advantages conferred by L455S.

Our results demonstrate that recurrent mutations in the RBD of BA.2.86 descendants can impact spike cleavage within virions. Specifically, JN.1 and KP.2 virions exhibit significantly reduced S1/(S1 + S) ratios compared to BA.2.86 or WA1, likely due to the shared L455S mutation in both spikes. In contrast, KP.3 virions maintain an S1/(S1 + S) ratio similar to that of BA.286 and WA1, suggesting that the unique Q493E mutation in KP.3 spike compensates for the reduced spike processing efficiency associated with L455S. Limited structural information is available around the furin cleavage site due to the high flexibility of this region. The cryogenic electron microscopy (cryo-EM) structure of the BA.2.86 spike has revealed some details of residues 621–640 surrounding the furin cleavage sites (36). In this structure, two RBD residues, T323 and Q321, form hydrogen bonds with Q628 and T630, respectively, suggesting an interaction between the RBD and the furin cleavage region. Mutations L455S and Q493E, although distant from the furin cleavage site, may indirectly affect these interactions by altering the RBD conformation, which could influence S1/S2 cleavage and S1 shedding within the virion. While the exact biological significance and underlying molecular mechanisms behind these phenotypes require further study, our findings suggest that these RBD mutations could influence viral infectivity through mechanisms beyond ACE2 binding, potentially by modifying spike processing (full-length or cleaved forms of spike) during virion maturation or secretion. The I670V mutation, identified in early BA.2.86 isolates but rare in later BA.2.86 or JN.1, is located near the furin cleavage site and does not contribute to immune evasion (28). The V1104L mutation in S2 may potentially stabilize the prefusion spike conformation (30). Neither I670V nor V1104L is likely to affect RBD binding or spike processing. However, their specific role in replication fitness remains to be elucidated.

In contrast to our findings, which generally align with epidemiological data, disparities in infectivity among BA.2.86-derived variants have been reported in pseudovirus systems. In particular, pseudotyped BA.2.86 and JN.1 spikes have shown inconsistent infectivity across various cell lines (30, 31). Pseudotyped KP.2 has demonstrated reduced infectivity compared to JN.1 (15, 28, 30), while pseudotyped KP.3 has been reported to have either higher infectivity than KP.2 (28) or comparable infectivities (18, 37). These discrepancies may stem from variations in cell lines, pseudovirus production, or experimental conditions. Our pairwise competition assay offers distinct advantages in comparing viral replication fitness. First, we use live-attenuated mNG SARS-CoV-2, which resembles authentic SARS-CoV-2 variants in structural characteristics, maturation, and infection pathways. Second, we utilize primary human airway epithelium cell cultures, a robust air-liquid interface model more relevant to respiratory SARS-CoV-2 infection than immortalized cell lines (26, 38). Third, by analyzing mixed viruses in pairwise competition experiments, we could minimize host-to-host variation, ensuring greater precision and reproducibility (39).

There are limitations to our study. We focused on the role of spike mutations in immune evasion and viral replication fitness among BA.2.86 descendants, leaving the influence of non-spike or synonymous mutations unexplored. Besides the spike mutations, wild-type KP.2 differs from JN.1 with a non-spike mutation, T1465I, in nonstructural protein 3 (NSP3), a multifunctional viral protein involved in polyprotein processing, replication complex formation, and antagonizing innate immunity (40). NSP3 is critical for viral fitness and virulence (41). Future mutagenesis studies using wild-type KP.2 could help pinpoint the potential contributions of NSP3 T1465I mutation to KP.2’s selective advantage over JN.1. Additionally, we did not investigate the roles of non-neutralizing antibodies and T cell immunity, despite their importance in protecting patients from severe disease and death (42, 43). Cellular immunity is known to be long-lasting and cross-reactive with Omicron spikes after vaccination or natural infection (44). Moreover, limited information was available regarding the infection history (when and what strains) before XBB.1.5 or JN.1 infection, vaccination (doses and type), or geographic or demographic factors. We also did not assess the baseline levels or longevity of humoral immunity. Despite these limitations, our study demonstrates that BA.2.86 descendants continue to evolve through genetic changes to fine-tune immune evasion and replication fitness, similar to earlier Omicron lineages. As of 12 October 2024, KP.3.1.1, a KP.3-derived sublineage with an S31 deletion in the spike, has become the predominant SARS-CoV-2 strain in the United States (5). Meanwhile, XEC, a hybrid of the Omicron lineages KS.1.1 and KP.3.3, has been increasingly detected worldwide (45). Therefore, it remains critical to continue monitoring the immune evasion and replication fitness of emerging SARS-CoV-2 variants to inform vaccine and monoclonal antibody design. Timely updates to countermeasures are essential to ensure adequate protection against new immune-evasive variants with enhanced fitness.

## Data Availability

All data supporting the findings have been included in this study. The sequence of SARS-CoV-2 strains can be accessed through GISAID (https://gisaid.org) with the following codes: BA.2.86 (EPI_ISL_18110065), JN.1 (EPI_ISL_18237538), KP.2 (EPI_ISL_ 19002640), and KP.3 (EPI_ISL_19016982). The sequence of SARS-CoV-2 mNG has been deposited in the supplemental information of our previous study (19).

## AFTER EPUB

*[This article was published on 29 April 2025 with some affiliation numbers missing in the byline. The affiliation designations were corrected in the current version, posted on 30 April 2025.]*

## SUPPLEMENTAL MATERIAL

Supplemental material mbio.03503-24-s0001.docx Supplemental figure and tables.

ASM does not own the copyrights to Supplemental Material that may be linked to, or accessed through, an article. The authors have granted ASM a non-exclusive, world-wide license to publish the Supplemental Material files. Please contact the corresponding author directly for reuse.
