# Supplementary material for: Comparative analysis of replication and immune evasion among SARS-CoV-2 subvariants BA.2.86, JN.1, KP.2, and KP.3
Source: mBio. 2025 Apr 29;16(6):e03503-24. doi: 10.1128/mbio.03503-24 (PMC12153290; doi:10.1128/mbio.03503-24)
Supplement: Supplemental material — Supplemental figure and tables. [file mbio.03503-24-s0001.docx]

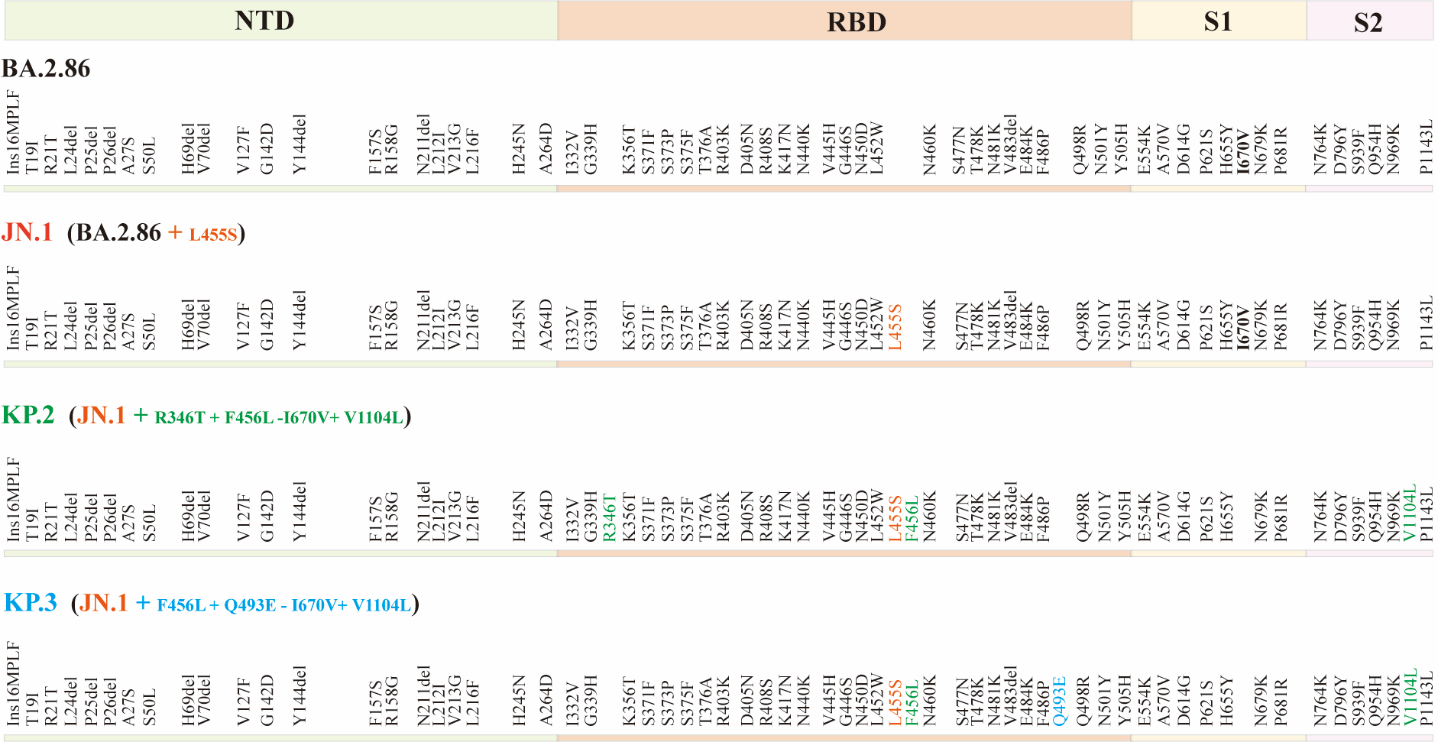


Figure S1. Spike mutations among BA.2.86 sublineage and its descendants. Amino acid changes in the BA.2.86 spike compared to the reference strain USA-WA1/2020 are shown in gray. Additional mutations in BA.2.86 descendants are color-coded as follows: green for R346T, F456L, and V1104L; orange for L455S; and blue for Q493E. The I670V mutation, present only in the BA.2.86 and JN.1 strains used in this study, is highlighted in bold. NTD: N-terminal domain of spike; RBD: receptor binding domain; S: spike glycoprotein; S1: N-terminal Furin cleavage fragment of S; S2: C-terminal Furin cleavage fragment of S.

Table S1. Sixty-one human serum samples collected 15-117 days after XBB.1.5-infection

| Serum ID | Age (year) | Gender (F/M) | Race or Ethnicity | Serum collection day (post-XBB.1.5 PCR^+^) | Serum collection date | Doses of parental mRNA vaccine plus BA.5 bivalent booster | *FFRNT_50_ | |
| --- | --- | --- | --- | --- | --- | --- | --- | --- |
|  |  |  |  |  |  |  | BA.2.86-spike | JN.1-spike |
| 1 | 42 | M | Black | 15 | 2/2/2023 | 3 doses | 3620 | 640 |
| 2 | 64 | M | White | 16 | 2/10/2023 | 2 doses | 1810 | 453 |
| 3 | 15 | F | Hispanic | 29 | 2/15/2023 | 2 doses | 320 | 320 |
| 4 | 27 | F | White | 31 | 2/24/2023 | 3 doses | 640 | 160 |
| 5 | 16 | M | Black | 39 | 2/27/2023 | 2 doses | 1280 | 320 |
| 6 | 38 | F | White | 19 | 3/3/2023 | 2 doses | 113 | 80 |
| 7 | 21 | F | White | 46 | 3/6/2023 | 3 doses | 113 | 20 |
| 8 | 46 | M | Hispanic | 35 | 3/6/2023 | 2 doses | 640 | 160 |
| 9 | 40 | F | Hispanic | 39 | 3/11/2023 | 2 doses | 905 | 160 |
| 10 | 56 | F | Black | 49 | 3/20/2023 | 3 doses | 320 | 160 |
| 11 | 42 | F | White | 25 | 3/22/2023 | 2 doses | 320 | 113 |
| 12 | 21 | M | White | 40 | 3/22/2023 | 2 doses | 160 | 80 |
| 13 | 36 | F | White | 19 | 3/22/2023 | 3 doses | 320 | 160 |
| 14 | 48 | F | White | 22 | 3/28/2023 | 2 doses | 640 | 320 |
| 15 | 30 | F | White | 54 | 3/28/2023 | 2 doses | 226 | 160 |
| 16 | 61 | F | Hispanic | 82 | 4/18/2023 | 3 doses | 40 | 57 |
| 17 | 25 | F | Hispanic | 42 | 4/18/2023 | 3 doses | 5120 | 640 |
| 18 | 34 | F | Black | 45 | 4/20/2023 | 2 doses | 160 | 160 |
| 19 | 53 | F | Hispanic | 73 | 4/27/2023 | 2 doses | 640 | 160 |
| 20 | 40 | F | Hispanic | 92 | 5/4/2023 | 2 doses | 113 | 40 |
| 21 | 26 | F | Hispanic | 48 | 5/4/2023 | 2 doses | 160 | 40 |
| 22 | 27 | F | Hispanic | 37 | 5/4/2023 | 2 doses | 1280 | 320 |
| 23 | 71 | M | Asian | 31 | 5/4/2023 | 2 doses | 1280 | 453 |
| 24 | 56 | F | Hispanic | 49 | 5/4/2023 | 3 doses | 160 | 80 |
| 25 | 47 | F | White | 64 | 5/5/2023 | 3 doses | 160 | 160 |
| 26 | 34 | F | White | 86 | 5/4/2023 | 3 doses | 160 | 160 |
| 27 | 35 | M | Asian | 72 | 5/5/2023 | 3 doses | 320 | 80 |
| 28 | 40 | F | Hispanic | 96 | 5/8/2023 | 2 doses | 20 | 20 |
| 29 | 39 | M | White | 65 | 5/9/2023 | 2 doses | 40 | 40 |
| 30 | 59 | F | Black | 42 | 5/9/2023 | 3 doses | 160 | 113 |
| 31 | 71 | F | White | 41 | 5/10/2023 | 2 doses | 7241 | 1280 |
| 32 | 47 | M | White | 63 | 5/12/2023 | 3 doses | 320 | 160 |
| 33 | 27 | F | Hispanic | 52 | 5/15/2023 | 2 doses | 453 | 320 |
| 34 | 56 | F | Hispanic | 81 | 5/15/2023 | 2 doses | 160 | 80 |
| 35 | 46 | F | White | 69 | 5/15/2023 | 2 doses | 1280 | 905 |
| 36 | 59 | F | White | 38 | 5/16/2023 | 3 doses | 1280 | 640 |
| 37 | 30 | M | White | 117 | 5/16/2023 | 3 doses | 320 | 80 |
| 38 | 56 | F | White | 62 | 5/16/2023 | 3 doses | 905 | 226 |
| 39 | 40 | F | Hispanic | 79 | 5/18/2023 | 4 doses | 640 | 226 |
| 40 | 20 | M | Black | 35 | 5/22/2023 | 2 doses | 5120 | 2560 |
| 41 | 28 | F | Asian | 75 | 5/22/2023 | 3 doses | 226 | 80 |
| 42 | 56 | M | Hispanic | 87 | 5/23/2023 | 2 doses | 1280 | 640 |
| 43 | 85 | F | White | 21 | 2/16/2023 | 4 doses + booster | 160 | 160 |
| 44 | 81 | F | White | 22 | 2/2/2023 | 4 doses + booster | 160 | 80 |
| 45 | 66 | F | Black | 20 | 2/3/2023 | 3 doses + booster | 640 | 160 |
| 46 | 65 | M | Hispanic | 17 | 2/17/2023 | 3 doses + booster | 2560 | 160 |
| 47 | 79 | F | Hispanic | 18 | 2/27/2023 | 3 doses + booster | 320 | 80 |
| 48 | 68 | F | White | 53 | 3/13/2023 | 3 doses + booster | 640 | 320 |
| 49 | 30 | F | Black | 52 | 3/15/2023 | 2 doses + booster | 160 | 80 |
| 50 | 79 | F | White | 50 | 3/17/2023 | 4 doses + booster | 640 | 226 |
| 51 | 75 | F | White | 22 | 3/28/2023 | 3 doses + booster | 640 | 905 |
| 52 | 71 | F | Black | 21 | 4/18/2023 | 4 doses + booster | 905 | 640 |
| 53 | 72 | M | White | 23 | 4/18/2023 | 4 doses + booster | 160 | 28 |
| 54 | 36 | F | Asian | 39 | 4/21/2023 | 3 doses + booster | 80 | 40 |
| 55 | 63 | F | White | 56 | 4/26/2023 | 3 doses + booster | 20 | 14 |
| 56 | 68 | M | White | 66 | 4/28/2023 | 4 doses + booster | 905 | 160 |
| 57 | 74 | M | White | 87 | 5/3/2023 | 4 doses + booster | 80 | 20 |
| 58 | 65 | M | White | 42 | 5/5/2023 | 3 doses + booster | 80 | 57 |
| 59 | 30 | F | Black | 103 | 5/5/2023 | 2 doses + booster | 113 | 40 |
| 60 | 29 | F | Hispanic | 42 | 5/16/2023 | 3 doses + booster | 640 | 113 |
| 61 | 43 | F | Hispanic | 51 | 5/18/2023 | 2 doses + booster | 226 | 40 |
| Median | 46 | - | - | 45 | - | - | - | - |
| ^#^GMT | - | - | - | - | - | - | 361 | 144 |
| ^†^95% CI | - | - | - | - | - | - | 260-501 | 109-192 |

*Individual FFRNT_50_ value is the geometric mean of duplicate FFRNT_50_ results.

^#^Geometric mean neutralizing titers (GMT).

^†^95% confidence interval (95% CI) for the GMT.

Table S2. Forty-one human serum samples collected 20-111 days after JN.1-infection

| ID | Age (year) | Gender  (F/M) | Race or Ethnicity | Serum collection day (post-JN.1 PCR^+^) | Serum collection date | *FFRNT_50_ | | |
| --- | --- | --- | --- | --- | --- | --- | --- | --- |
|  |  |  |  |  |  | JN.1-spike | KP.2-spike | KP.3-spike |
| 1 | 52 | F | Black | 21 | 12/28/2023 | 320 | 160 | 160 |
| 2 | 63 | F | White | 25 | 1/4/2024 | 113 | 40 | 28 |
| 3 | 84 | F | Black | 26 | 1/2/2024 | 320 | 160 | 226 |
| 4 | 28 | F | White | 33 | 12/26/2023 | 320 | 113 | 226 |
| 5 | 33 | F | Hispanic | 26 | 1/3/2024 | 640 | 320 | 453 |
| 6 | 66 | F | White | 23 | 1/9/2024 | 113 | 40 | 20 |
| 7 | 50 | F | Hispanic | 47 | 12/31/2023 | 28 | 40 | 40 |
| 8 | 30 | F | White | 45 | 1/5/2024 | 905 | 320 | 226 |
| 9 | 55 | F | Hispanic | 51 | 12/31/2024 | 160 | 80 | 80 |
| 10 | 34 | F | White | 30 | 1/22/2024 | 1280 | 640 | 453 |
| 11 | 25 | F | Hispanic | 34 | 1/18/2024 | 320 | 113 | 160 |
| 12 | 51 | F | Hispanic | 28 | 1/25/2024 | 905 | 320 | 640 |
| 13 | 40 | F | Hispanic | 20 | 2/7/2024 | 2560 | 1280 | 1280 |
| 14 | 40 | F | Hispanic | 63 | 12/29/2023 | 80 | 80 | 113 |
| 15 | 23 | F | Black | 47 | 1/19/2024 | 320 | 113 | 160 |
| 16 | 59 | F | White | 26 | 2/9/2024 | 640 | 320 | 320 |
| 17 | 7 | F | White | 58 | 1/8/2024 | 1280 | 320 | 640 |
| 18 | 67 | F | White | 49 | 1/18/2024 | 226 | 160 | 160 |
| 19 | 35 | F | White | 38 | 1/30/2024 | 640 | 160 | 160 |
| 20 | 73 | F | White | 57 | 1/11/2024 | 453 | 113 | 160 |
| 21 | 57 | F | Black | 33 | 2/6/2024 | 1810 | 905 | 1280 |
| 22 | 46 | F | White | 55 | 1/15/2024 | 2560 | 1280 | 905 |
| 23 | 80 | F | White | 35 | 2/5/2024 | 160 | 20 | 20 |
| 24 | 40 | F | Hispanic | 30 | 2/12/2024 | 1810 | 320 | 160 |
| 25 | 16 | F | Hispanic | 45 | 1/29/2024 | 160 | 80 | 80 |
| 26 | 38 | F | Hispanic | 37 | 2/12/2024 | 40 | 20 | 10 |
| 27 | 40 | F | Black | 44 | 2/13/2024 | 640 | 160 | 160 |
| 28 | 42 | F | White | 33 | 2/26/2024 | 160 | 80 | 80 |
| 29 | 67 | F | White | 68 | 1/25/2024 | 226 | 80 | 226 |
| 30 | 56 | F | Hispanic | 42 | 2/20/2024 | 80 | 40 | 80 |
| 31 | 70 | F | White | 54 | 2/8/2024 | 453 | 57 | 160 |
| 32 | 27 | F | White | 57 | 2/8/2024 | 160 | 160 | 160 |
| 33 | 44 | M | Hispanic | 90 | 1/5/2024 | 453 | 320 | 453 |
| 34 | 59 | F | Black | 57 | 2/7/2024 | 640 | 453 | 640 |
| 35 | 39 | F | Hispanic | 47 | 2/21/2024 | 640 | 640 | 640 |
| 36 | 67 | M | Hispanic | 86 | 1/22/2024 | 905 | 226 | 320 |
| 37 | 76 | F | White | 86 | 1/22/2024 | 5120 | 2560 | 1280 |
| 38 | 77 | M | Black | 56 | 2/21/2024 | 5120 | 640 | 320 |
| 39 | 18 | M | Hispanic | 68 | 2/20/2024 | 320 | 160 | 160 |
| 40 | 66 | F | White | 111 | 1/18/2024 | 226 | 80 | 160 |
| 41 | 38 | F | White | 84 | 2/15/2024 | 3620 | 320 | 320 |
| Median | 46 | - | - | 45 | - | - | - | - |
| ^#^GMT | - | - | - | - | - | 437 | 174 | 193 |
| ^†^95% CI | - | - | - | - | - | 296-646 | 122-248 | 135-275 |

*Individual FFRNT_50_ value is the geometric mean of duplicate FFRNT_50_ results.

^#^Geometric mean neutralizing titers (GMT).

^†^95% confidence interval (95% CI) for the GMT.
